# Supplementary material for: Physical Engagement in Face-to-Face Interaction Is Associated with Depressive Symptoms of Interaction Partners in the Workplace
Source: Behav Sci (Basel). 2024 Oct 30;14(11):1006. doi: 10.3390/bs14111006 (PMC11591311; doi:10.3390/bs14111006)
Supplement: Supplementary file 1 [file behavsci-14-01006-s001.zip › behavsci-3203489-supplementary.pdf]

Supplementary information

Physical engagement in face-to-face interaction is associated with depressive symptoms of interaction partners in the workplace

Jong-Hyeok Lee[1,*], Nobuo Sato[2], Taiki Ogata[1], Kazuo Yano[1,2,3], Yoshihiro Miyake[1]

[1]Department of Computer Science, Tokyo Institute of Technology, Yokohama, 226-8502, Japan

[2]Happiness Planet, Ltd., Kokubunji, Tokyo 185-8601, Japan

[3]Hitachi, Ltd., Kokubunji, Tokyo, 185-8601, Japan

[*]Corresponding author: mons1220@gmail.com

Supplementary Note S1

In this study, we defined the baseline body rhythm as the average body rhythm during the 30 min preceding an interaction, referred to as the baseline window span. To evaluate the potential impact of this methodological choice on our results, we investigated how varying the baseline window span from 10 to 60 minutes affected our findings.

We examined the correlations between physical engagement and depression scores across different baseline window spans (Fig. S1). And our main findings remained statistically significant ($p < 0.001$) across the range of baseline windows from 10 to 60 minutes:

1. The correlation between physical engagement and depression score (partner)
2. The correlation between physical engagement (partner) and depression score (partner)

This consistency demonstrates the robustness of our main findings, indicating that they are not artifacts of our specific choice of a 30 min baseline window span.

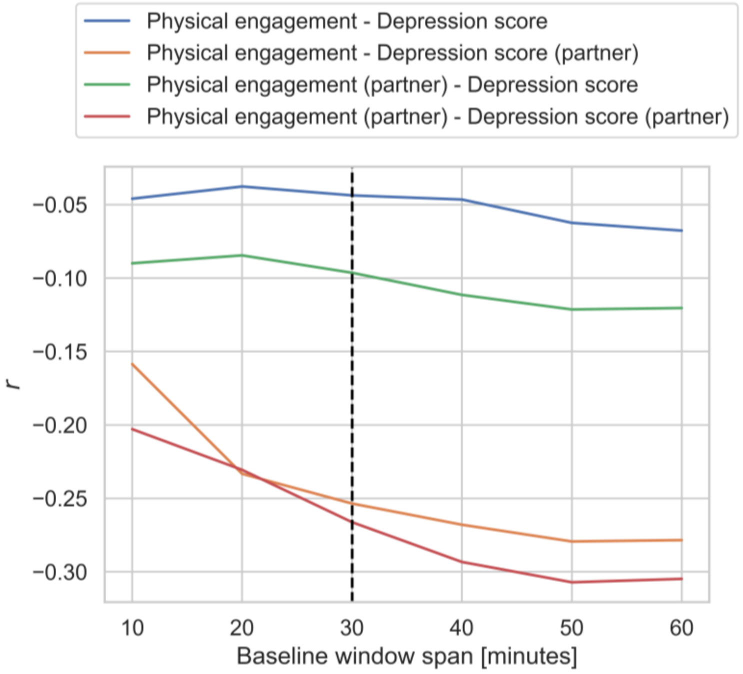

Figure S1. Correlations between physical engagement and depression scores of employees and their partners based on different baseline window spans. The black dashed line represents the 30-minute baseline window span established in this study.

Supplementary Note S2

To more accurately define the body rhythm increase induced by face-to-face interactions, we examined the average changes in body rhythm observed before and after face-to-face interactions (Fig. S2). The results show that the impact of face-to-face interactions on body rhythm extends approximately 10 minutes before and after the interaction. These effects might include misalignment between wearable devices, physical activities due to mutual recognition (such as greetings), and contexts like movement or preparation before interactions. However, we have already identified and corrected the possibility of intermittent misalignment during data pre-processing. Misalignment issues could have significant implications in this study since we use the difference in body rhythm before and during interaction to calculate physical engagement. Therefore, we determined that the interaction's influence exists within a broader window where its effects on body rhythm are confirmed. Detailed methodology is addressed in the Methods section.

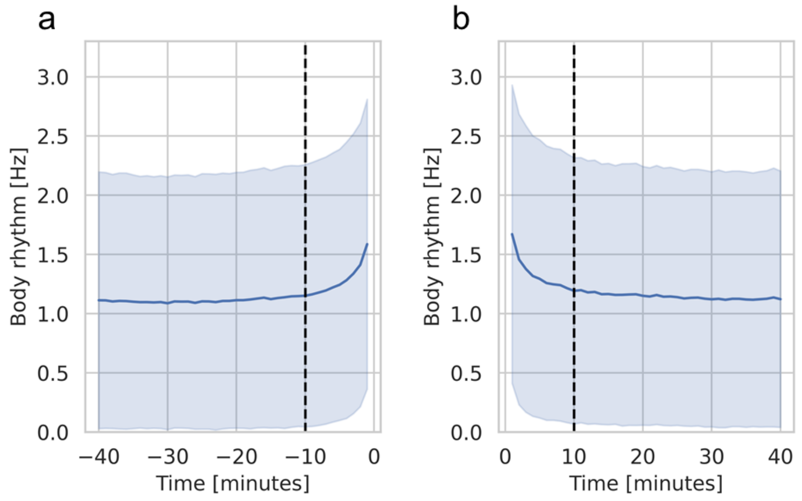

Figure S2. Body rhythm before (a) and after (b) face-to-face interaction logs. For all observed body rhythms before and after face-to-face interaction logs, solid lines represent mean values, and shaded areas indicate standard deviations. Dashed lines in (a) and (b) denote the time points that are 10 minutes before and after the interaction, respectively.

Supplementary Note S3

Figure S3 provides visual representations of the distribution of increased body rhythm during face-to-face interactions compared to baseline, which is defined as physical engagement.

Figure S3a shows a histogram of the data, displaying an approximately bell-shaped distribution consistent with the reported mean (0.26) and median (0.24). The near-symmetry of the distribution is visually evident, aligning with the reported skewness (0.00896).

Figure S3b presents a Q-Q plot, comparing sample quantiles with theoretical quantiles from a normal distribution. The plot demonstrates a strong linear relationship, particularly in the central region, suggesting a close approximation to normality. Slight deviations at the extremes suggest marginally thicker tails, consistent with the reported kurtosis (0.97).

Together, these visualizations corroborate the numerical summary statistics and support the assumption that the distribution of body rhythm increase sufficiently approximates normality.

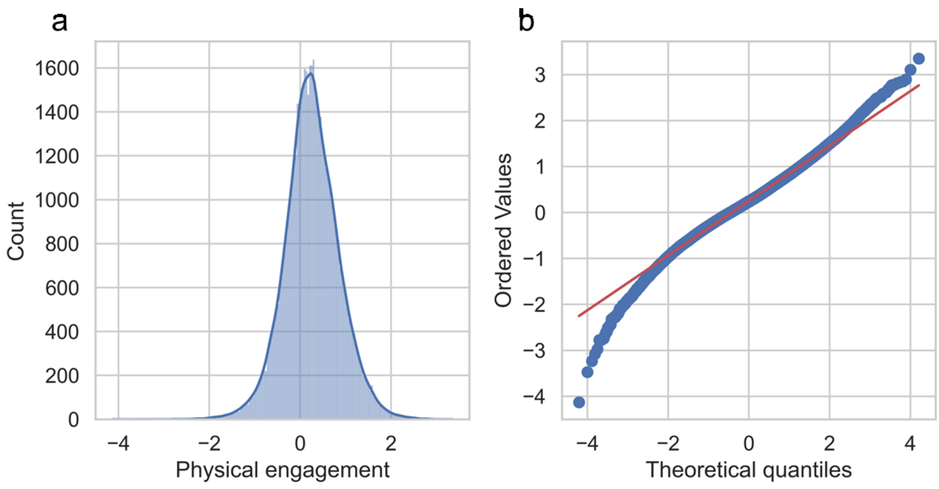

Figure S3. Distribution of increased body rhythm (physical engagement) during face-to-face interactions compared to baseline (a), and Q-Q plot comparing the sample quantiles of increased body rhythm to the theoretical quantiles of a normal distribution (b).

Supplementary Note S4

To verify the robustness of our primary results (Fig. 2a-d) against outliers or non-linearity, we conducted Spearmans correlation analyses to examine the association between physical engagement and depression scores among employees and their face-to-face interaction partners.

1. The correlation between physical engagement and depression score: $r = -0.04$, $p = 0.42$
2. The correlation between physical engagement and depression score (partner): $r = -0.26$, $p < 0.001$
3. The correlation between physical engagement (partner) and depression score: $r = -0.08$, $p = 0.08$
4. The correlation between physical engagement (partner) and depression score (partner) : $r = -0.25$, $p < 0.001$

The results were consistent with our Pearson correlation analyses, demonstrating that our main findings are robust to outliers and potential non-linear relationships.
